# The oxidative stress-inflammation self-perpetuating cycle in multiple sclerosis: from mechanisms to emerging antioxidant strategies

**DOI:** 10.3389/fphar.2026.1806176

**Published:** 2026-04-20

**Authors:** Baiyang Yuan, Xuan Jin, Chunmei Li, Yifeng Shen, Jingwei Tian, Sijin Duan

**Affiliations:** 1 School of Pharmacy, Key Laboratory of Molecular Pharmacology and Drug Evaluation (Yantai University), Ministry of Education, Collaborative Innovation Center of Advanced Drug Delivery System and Biotech Drugs in Universities of Shandong, Yantai University, Yantai, China; 2 Shanghai Mental Health Center, School of Medicine, Shanghai Jiao Tong University, Shanghai, China

**Keywords:** antioxidants, multiple sclerosis, oxidative stress, pathogenesis, treatment

## Abstract

Multiple sclerosis (MS) is a complex multifactorial disease of the central nervous system (CNS) whose pathogenesis has not yet been fully elucidated. Current MS treatments primarily consist of disease-modifying therapies with anti-inflammatory and immunomodulatory properties, which effectively reduce relapse rates and disease activity. However, these therapies often exhibit limited long-term efficacy, may cause severe adverse effects, and remain largely insufficient in preventing the progressive accumulation of irreversible disability driven by axonal and neuronal damage. Although oxidative stress (OS) is not the sole pathologic factor in MS, substantial evidence supports its critical contribution to disease development and progression. In particular, OS is closely associated with key pathological processes such as demyelination and axonal degeneration. OS can act as a signaling mediator that promotes inflammatory responses, while inflammatory processes further amplify OS, forming a self-perpetuating cycle that exacerbates CNS tissue injury. Consequently, increasing attention has been directed toward the development of antioxidant-based therapeutic strategies for MS. Nevertheless, a comprehensive synthesis of MS drug development from the perspective of antioxidant capacity remains lacking, limiting rational therapeutic. This review examines the interplay between inflammation and OS in MS pathology, and summarizes current advances in antioxidant-based therapeutic approaches. By integrating existing evidence, this work aims to clarify the role of OS in MS pathogenesis and to inform the development of effective antioxidant-oriented treatments.

## Highlights

Literature searches were performed in PubMed and Web of Science to identify relevant articles published up to January 2026. The search strategy combined terms related to multiple sclerosis and redox biology, including “multiple sclerosis”, “oxidative stress”, “reactive oxygen species”, “neuroinflammation”, “NRF2”, “dimethyl fumarate”, “progressive multiple sclerosis”, and “antioxidant therapy”.

## 1 Introduction

Multiple sclerosis (MS) is a chronic inflammatory disease of the central nervous system (CNS) characterized by immune-mediated demyelination accompanied by axonal and neuronal damage (Marcus, 2022). The global prevalence of MS is estimated at 50-300 cases per 100,000 individuals, with a markedly higher incidence in women (female-to-male ratio of approximately 2.4) (Galea et al., 2015). MS most commonly affects young adults between 20 and 40 years of age and represents a leading cause of acquired, non-traumatic neurological disability in this population (Naseri et al., 2021; Scalfari et al., 2011). Although the etiology of MS remains incompletely understood, accumulating evidence suggests that disease development arises from the interplay between genetic susceptibility and environmental factors. Genetic risk is strongly associated with specific loci, most notably HLA-DRB1 (International Multiple Sclerosis Genetics C, 2019). In addition, immune tolerance dysfunction driven by environmental and lifestyle factors (cigarette smoking, air pollution, Epstein-Barr virus infection (Soldan and Lieberman, 2023), nutritional status, vitamin D deficiency (Jagannath et al., 2018), and alterations in gut microbiota) plays a critical role in disease initiation and progression (Marcus, 2022; Dobson and Giovannoni, 2019).

Clinically, MS affects both the brain and spinal cord and is categorized into distinct phenotypic subtypes: relapsing-remitting MS (RRMS), primary progressive MS (PPMS), and secondary progressive MS (SPMS) (Marcus, 2022). The majority of patients initially present with RRMS, characterized by acute or subacute episodes of neurological dysfunction, such as visual impairment, sensory or motor deficits, ataxia, or bladder dysfunction. These episodes are followed by periods of partial or complete remission. Overtime, however, many patients experience a transition to SPMS, in which relapse frequency declines while irreversible neurological disability progressively accumulates (Klotz et al., 2023; Compston and Coles, 2008; Filippi et al., 2018). In contrast, PPMS is defined by a gradual and continuous worsening of neurological function from disease onset, without distinct relapses or remissions. These temporal differences between relapsing and progressive MS are therapeutically important, as the relative contributions of acute inflammation, chronic neurodegeneration, and oxidative injury may vary across disease stages, thereby influencing the timing and potential efficacy of antioxidant interventions.

Since interferon-β (IFN-β) was approved for the treatment of RRMS in 1993, substantial progress has been made in targeting peripheral immune-driven inflammatory processes through a range of anti-inflammatory and immunomodulatory therapies (Koch-Henriksen and Sorensen, 2000). Despite these advances, current treatments often exhibit limited long-term efficacy and may be associated with significant adverse effects. Moreover, they remain largely ineffective in preventing the progressive accumulation of irreversible disability resulting from axonal and neuronal damage, particularly during the progressive stages of the disease (Correale et al., 2019; Singh et al., 2017; Confavreux et al., 2000). Consequently, the development of novel neuroprotective therapies aimed at preventing or delaying neurological deterioration represents a critical unmet need in MS management (Filippi and Rocca, 2020). Oxidative stress (OS) has emerged as an important contributor to both MS pathogenesis and, critically, disease progression (Sanabria-Castro et al., 2024). Elevated levels of OS, together with dysregulated glutamate metabolism, promote axonal degeneration (Haider, 2015; Ohl et al., 2016), and are closely associated with disease relapse and progression. Accordingly, the pleiotropic mechanisms of antioxidants-including mitigation of oxidative damage, modulation of immune responses, and support of neural repair pathways observed in preclinical models provide a strong rationale for investigating them as potential disease-modifying strategies in MS. However, it is critical to distinguish this mechanistic rationale from established clinical efficacy. To date, dimethyl fumarate (DMF) remains the primary example for which antioxidant properties have translated into proven therapeutic benefit in MS; the clinical relevance of most other antioxidant approaches is yet to be definitively established. In this review, we integrate current evidence on the interplay between inflammation and OS in MS pathology and critically assess recent advances in antioxidant-oriented therapeutic strategies with the potential to improve MS treatment outcomes.

## 2 OS in MS

### 2.1 OS

Mitochondria are the principal intracellular source of reactive oxygen species (ROS). Beyond their role as the cellular “powerhouse”, mitochondria participate in numerous redox reactions (Peoples et al., 2019) and generate adenosine triphosphate (ATP) essential for cellular homeostasis (Chandel, 2021). Four major forms of ROS are commonly recognized: superoxide anion (O_2_^•-^), hydrogen peroxide (H_2_O_2_), hydroxyl radical (•OH), and singlet oxygen (^1^O_2_) (Kracun et al., 2025). Under physiological conditions, cells maintain redox homeostasis through multiple antioxidant defense mechanisms that efficiently scavenge ROS. Importantly, ROS are not solely detrimental by-products of cellular metabolism. At low to moderate concentrations, ROS function as second messengers in redox signaling and are essential for normal cellular physiology (Sies and Jones, 2020). Spatially and temporally controlled ROS production regulates diverse processes, including cell proliferation, differentiation, adaptation to hypoxia, autophagy, and immune defense. In CNS, physiological ROS signaling contributes to synaptic plasticity, neuronal survival, and intracellular communication, highlighting that redox balance, rather than complete ROS elimination, is critical for cellular homeostasis (Sies and Jones, 2020). In this context, redox signaling should be distinguished from oxidative damage. Redox signaling generally refers to reversible and selective oxidation of redox-sensitive molecular targets, such as cysteine residues in signaling proteins, thereby modulating downstream pathways in a controlled manner (Finkel, 2011). By contrast, oxidative damage occurs when ROS generation exceeds antioxidant buffering capacity, leading to non-selective and often irreversible oxidation of lipids, proteins, and nucleic acids. Thus, the biological consequences of ROS depend not only on their abundance, but also on their species, subcellular origin, duration of exposure, and local antioxidant capacity (Sies and Jones, 2020).

**FIGURE 1:**
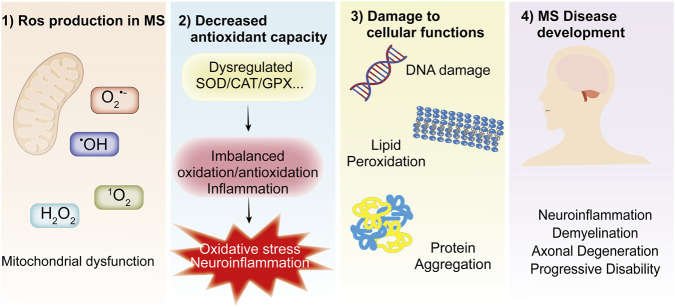
The Central Role of OS in Driving Disease Initiation and Progression. (1) Mitochondria are the primary site of intracellular reactive oxygen species (ROS) generation, producing superoxide anion (O_2_^•-^), hydroxyl radical (•OH), hydrogen peroxide (H_2_O_2_), and singlet oxygen (^1^O_2_). (2) Dysregulation of antioxidant enzymes (SOD, CAT, GPX) disrupts redox homeostasis, triggering OS via an imbalance between pro-oxidant and antioxidant pathways. (3) This stress induces structural and functional damage to DNA, lipid membranes, and proteins, (4) which in turn contributes to the development of neurodegenerative diseases, cancer, and cardiovascular pathologies.

In contrast, exposure to diverse physiological or pathological stressors can lead to excessive ROS production, resulting in OS. OS damages critical cellular components, including DNA, lipid membranes, and proteins, thereby impairing cellular structure and function (Kracun et al., 2025). In addition to ROS, reactive nitrogen species (RNS) including nitric oxide (NO) and peroxynitrite (ONOO^−^) are key mediators of nitrosative stress in MS pathology (Li et al., 2023). RNS are primarily generated by the enzymatic activity of inducible nitric oxide synthase (iNOS), which is substantially upregulated in active MS lesions within macrophages, microglia, and astrocytes (Hill et al., 2004). NO itself can exert both beneficial and detrimental effects depending on its concentration and cellular context. However, when produced in excess, NO rapidly reacts with O_2_^•-^ to form ONOO^−^, a highly reactive oxidant that promotes protein nitration, lipid peroxidation, mitochondrial dysfunction, and axonal damage (Ohl et al., 2016). Peroxynitrite-mediated nitration of tyrosine residues on proteins orming 3-nitrotyrosine has been documented in active MS plaques and correlates with disease severity and neurodegeneration (Hill et al., 2004; Wu et al., 2024). Thus, both ROS and RNS contribute to the oxidative/nitrosative stress imbalance that amplifies tissue injury and disease progression. OS typically arises from a disruption of the oxidant/antioxidant balance and can drive both neuroinflammatory and neurodegenerative processes, including those observed in MS (Escribano et al., 2017; Kracun et al., 2025) (Figure 1). This imbalance is characterized by a self-reinforcing interplay between increased levels of ROS, pro-inflammatory transcription factors, enzymatic oxidants, and oxidative end products, accompanied by reduced activity of antioxidant transcription factors and diminished enzymatic and non-enzymatic antioxidant defenses. This dual dysregulation enhanced oxidant production coupled with impaired antioxidant capacity amplifies neuroinflammation and promotes neurodegeneration in MS (Tonev and Momchilova, 2023).

### 2.2 OS markers in MS

Although the precise etiology of MS remains incompletely understood, OS is widely recognized as a pivotal contributor to disease pathogenesis (Ohl et al., 2016; Tobore, 2021; Pegoretti et al., 2020). Systematic investigation of OS-related biomarkers in patients with MS may therefore provide valuable insights into disease mechanisms, as well as potential diagnostic and therapeutic implications. In active MS lesions, oligodendrocytes and neurons exhibit marked oxidative damage, characterized by accumulation of 8-hydroxy-2′-deoxyguanosine (8-OHdG), a specific marker of DNA oxidation, and increased immunoreactivity for lipid peroxidation products such as HNE; this oxidative injury is most pronounced in areas of active demyelination and directly correlates with cell death and neurodegeneration (Haider et al., 2011). Evidence of enhanced lipid peroxidation has been detected in both the cerebrospinal fluid (Hunter et al., 1985) and plasma (Naidoo and Knapp, 1992) of patients with MS, and multiple lipid peroxidation products have been identified within active MS plaques and other affected brain regions (Haider et al., 2011). Malondialdehyde (MDA), a terminal product of the oxidative degradation of polyunsaturated fatty acids (PUFAs), is widely used as a biomarker of lipid peroxidation (Gueraud et al., 2015). Elevated levels of isoprostanes and MDA have been reported in the plasma of MS patients compared with healthy controls, supporting their potential utility as indicators of disease development, disability progression, or treatment response (Ibitoye et al., 2016). Among lipid peroxidation markers, isoprostanes are considered one of the most reliable measures of *in vivo* OS status (Abramova et al., 2024). These lipid peroxidation products are not merely passive biomarkers; they actively propagate oxidative injury by reacting with proteins and DNA, forming adducts that impair cellular function and amplify inflammatory responses, thereby perpetuating the OS-inflammation cycle (Gueraud et al., 2015).

In parallel, alterations in endogenous antioxidant defense systems have been consistently observed in MS. Key cytoprotective enzymes and molecules involved in ROS scavenging, including superoxide dismutase (SOD), glutathione peroxidase (GPx), catalase (CAT), and glutathione (GSH), play central roles in maintaining redox homeostasis. Bizoń et al. reported that in patients with RRMS, SOD activity did not differ significantly from that in healthy controls (Bizon et al., 2023), whereas GPx activity was significantly reduced and CAT activity was significantly increased (Bizon et al., 2023). The reduction in GPx activity suggests impaired capacity to detoxify lipid hydroperoxides, directly contributing to the accumulation of oxidative damage, while increased CAT activity may represent a compensatory response to elevated H_2_O_2_ levels. Other studies have demonstrated elevated activities of SOD1 and SOD2 within active demyelinating lesions (Kemp et al., 2016) and in the cerebellar gray matter of MS patients (van Horssen et al., 2008), as well as increased CAT activity in active lesions (van Horssen et al., 2008). The upregulation of SOD within lesions likely reflects an adaptive response to increased superoxide production, but if this is not matched by commensurate increases in GPx and CAT, it may paradoxically lead to H_2_O_2_ accumulation and exacerbate OS (van Horssen et al., 2008). Collectively, these findings indicate a dysregulated antioxidant enzyme system in MS, with enzyme activity levels closely linked to disease course and severity. Proton magnetic resonance spectroscopy (^1^HMRS) studies have further revealed reduced GSH levels in specific brain regions of MS patients (Choi et al., 2011; Choi et al., 2017), providing important metabolic evidence for the involvement of OS in disease pathology. GSH, the predominant intracellular antioxidant, protects against oxidative damage and peroxide-induced toxicity through the catalytic actions of glutathione S-transferase (GST) and GPx (Maiorino et al., 2015); its depletion in MS directly compromises the cellular capacity to neutralize ROS and detoxify electrophiles, rendering oligodendrocytes and neurons particularly vulnerable to oxidative injury. GSH, the predominant intracellular antioxidant, protects against oxidative damage and peroxide-induced toxicity primarily through the catalytic actions of GST and GPx (Maiorino et al., 2015). Consistent with this role, GPx activity has been reported to be significantly decreased in the CSF (Calabrese et al., 1994), serum (Bizon et al., 2023), plasma (Tasset et al., 2012) and leukocytes (Naziroglu et al., 2014) of MS patients compared with controls. This systemic reduction in GPx activity further underscores the widespread nature of antioxidant defense impairment in MS, extending beyond the CNS to peripheral compartments.

Beyond classical antioxidant enzymes, dysregulation of multiple OS-related proteins and pathways has been reported in MS, including peroxiredoxin (PRX), an enzyme critical for reducing H_2_O_2_ whose dysregulation may impair the cellular ability to handle peroxides (Voigt et al., 2017; Ovchinnikova et al., 2024), DJ-1 protein, a redox-sensitive chaperone and sensor of OS whose alteration reflects an adaptive response to oxidative challenge (van Horssen et al., 2010), and myeloperoxidase (MPO), an enzyme expressed by activated microglia and macrophages that generates hypochlorous acid and contributes directly to oxidative tissue damage (Gray et al., 2008). In addition, ferroptosis, a distinct form of regulated cell death characterized by iron-dependent lipid peroxidation has been increasingly implicated in MS pathology. Ferroptosis represents a convergence point where OS, iron accumulation, and lipid peroxidation intersect to drive cell death, providing a mechanistic link between OS and oligodendrocyte loss in MS lesions (Luoqian et al., 2022). Evidence suggests enhanced ferroptotic activity within active and chronic MS lesions, as well as in the CSF of MS patients, identifying ferroptosis as a potentially pathogenic and modifiable process in MS (Luoqian et al., 2022; Van San et al., 2023). Genome-wide association studies (GWAS) have further identified more than 200 genetic loci associated with MS susceptibility, including several genes involved in mitochondrial function and OS defense (UCP3, GRPEL1, TXNRD2, ISCU, AASS, ACADL, DMGDH, and CADS) (International Multiple Sclerosis Genetics C, 2019; Kim and Patsopoulos, 2022; Fischer et al., 2012). The association of these variants with MS susceptibility suggests that genetic predisposition to impaired OS defense may contribute to disease risk. Taken together, these findings suggest that within and surrounding MS lesions, oxidative damage is accompanied by a compensatory antioxidant stress response aimed at counteracting excessive ROS accumulation (van Horssen et al., 2008; Haider et al., 2011). However, the long-term consequences of this response and its causal relationship to disease progression remain incompletely understood and warrant further investigation (Bellisario et al., 2025; Kamarehei and Zahednasab, 2025).

### 2.3 The inflammatory-oxidative axis in MS

**FIGURE 2:**
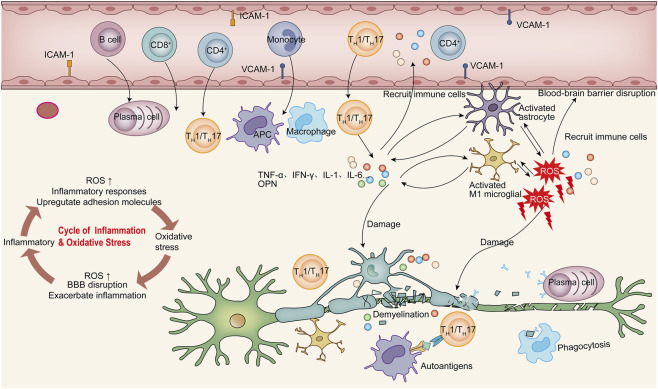
A Self-Perpetuating Cycle of OS and Inflammation Drives MS. Genetic susceptibility and environmental triggers activate peripheral lymphocytes, including CD4^+^ (T_H_1/T_H_17 subsets), CD8^+^ T cells, and B cells. These immune cells cross the BBB via interactions between leukocyte α4-integrin and endothelial adhesion molecules (ICAM-1, VCAM-1). Within the CNS, infiltrating immune cells and activated resident glia (microglia, astrocytes) release pro-inflammatory cytokines (TNF-α, IFN-γ, IL-1, IL-6) and ROS. This triggers oxidative stress and a self-reinforcing inflammatory cycle: ROS disrupts BBB integrity to enhance leukocyte infiltration, while inflammation further elevates ROS production. OS and inflammation directly damage oligodendrocytes, causing demyelination, and impair mitochondrial function in axons, exacerbating ROS generation and axonal degeneration. Activated microglia phagocytose myelin debris, and astrocytes secrete CSPGs to form glial scars, inhibiting remyelination. The cycle perpetuates as myelin autoantigens re-activate infiltrating T cells, sustaining inflammation and OS injury, which ultimately drives neurodegeneration in MS.

In MS pathogenesis, the inflammation-driven oxidative burst generated by activated microglia, astrocytes, and infiltrating macrophages play a critical role in demyelination and subsequent injury to neurons, axons, myelin, and oligodendrocytes (Bo et al., 1994; Ohl et al., 2016) (Figure 2). Genetic susceptibility and environmental exposures promote early activation of lymphocytes in the peripheral immune system (Beecham et al., 2013). Activated CD4^+^ and CD8^+^ T cells, including T helper 1 (T_H_1) and T_H_17 subsets, together with B cells and innate immune cells, subsequently cross the blood-brain barrier (BBB) and infiltrate the CNS. This proess is mediated in part by interactions between surface α4-integrin expressed on leukocytes and cell adhesion molecules (CAMs) on vascular endothelial cells. Once within the CNS, these immune cells initiate myelin-specific immune responses, leading to inflammation and tissue injury (Steinman, 2009; Takeshita and Ransohoff, 2012). Among CD4^+^ T cell populations, T_H_1 and T_H_17 cells are particularly implicated in MS pathogenesis and exhibit elevated expression of their signature cytokines, IFN-γ and IL-17A, respectively (Cao et al., 2015).

As the disease progresses, diffuse infiltration of inflammatory T cell occurs, accompanied by the release of pro-inflammatory cytokines such as TNF-α, IL-1 and IL-6. These mediators enhance the cytotoxic potential of resident glial cells, including microglia and astrocytes (Dendrou et al., 2015). Microglia, the primary innate immune sentinels of the CNS, rapidly respond to inflammatory cues and can transition into distinct activation states, traditionally categorized as the neurotoxic M1 phenotype (associated with pro-inflammatory cytokine release) or the neuroprotective M2 phenotype (involved in tissue repair and inflammation resolution) (Hammond et al., 2019; Kwon and Koh, 2020). In M1-activated microglia, the respiratory burst system represents a major source of ROS. Excessive production of ROS, together with pro-inflammatory cytokines and chemokines, amplifies inflammatory signaling and exacerbates CNS injury (Lee and Yoo, 2025). Concurrently, activated astrocytes and oligodendrocytes secrete chondroitin sulfate proteoglycans (CSPGs), contributing to glial scar formation, which represents a major barrier to axonal regeneration and remyelination in MS (Keough et al., 2016). Oligodendrocytes are essential for the generation and maintenance of the myelin sheath and for axonal metabolic support (Simons and Nave, 2015). Inflammatory mediators and OS directly damage oligodendrocytes, leading to demyelination and disruption of saltatory conduction (Franklin and Simons, 2022). This process results in widespread myelin loss and axonal injury, and ultimately contributes to neurodegeneration (Jakel et al., 2019). Demyelination is a defining pathological hallmark of MS, and myelin-associated proteins are thought to serve as primary autoreactive targets (Kaushansky et al., 2010). Within the CNS, infiltrating CD4^+^ T cells undergo antigen-driven re-activation in response to these targets, further perpetuating inflammatory cascades (Goverman, 2009).

Mitochondrial dysfunction represents another key mechanism linking inflammation and OS in MS. Excessive ROS can induce lipid peroxidation of fatty acids localized near mitochondria, generating reactive peroxides that exert lipotoxic effects on mitochondrial DNA (mtDNA), RNA, and proteins, thereby impairing mitochondrial function (Chen et al., 2024). Mitochondrial dysfunction, in turn, enhances ROS generation, creating a feed-forward loop that exacerbates OS. Such mitochondrial injury has been implicated in characteristic MS lesion pathology, including demyelination, oligodendrocyte apoptosis, and axonal degeneration (Zorov et al., 2014; Lassmann et al., 2012). In axons, impaired mitochondrial ATP production disrupts ionic homeostasis across intracellular and extracellular compartments, ultimately leading to axonal degeneration and neuronal cell death (Trapp and Stys, 2009).

During lesion progression, activated microglia phagocytose iron released from degenerating oligodendrocytes, a process that may further amplify oxidative tissue damage (Lassmann, 2014). In parallel, depletion of mtDNA in cortical neurons (Campbell et al., 2011), combined with iron accumulation in oligodendrocytes, can intensify neuronal OS driven by inflammation and mitochondrial dysfunction, thereby accelerating axonal degeneration (Lassmann et al., 2012).

Inflammation and OS are thus tightly interconnected processes in MS, forming a mutually reinforcing and self-perpetuating pathological cycle. Accordingly, OS in MS should be regarded not merely as a secondary byproduct of inflammation, but also as an active driver of demyelination, axonal injury, and lesion evolution. Elevated levels of oxygen-derived free radicals can disrupt BBB integrity, enhance leukocytes infiltration, and directly mediate demyelination and axonal injury, thereby exacerbating autoimmune inflammation through multiple signaling pathways (Kim et al., 2024; Zhao et al., 2025). Although numerous molecular pathways have been implicated in this crosstalk, the dominant signaling mechanisms remain incompletely defined (Lei and Lin, 2024). It has been shown that excessive ROS may promote neuroinflammation and tissue damage by enhancing T-cell activity via the arachidonic acid cascade and by directly or indirectly impairing the integrity of BBB and myelin (Cooper, 1997). Moreover, ROS can activate redox-sensitive transcription factors, such as nuclear transcription factor-kappa B (NF-κB), leading to upregulation of key pro-inflammatory mediators and adhesion molecules implicated in experimental autoimmune encephalomyelitis (EAE, the most commonly used animal model of MS) and MS, including tumor necrosis factor-α (TNF-α), inducible iNOS, intracellular adhesion molecule 1 (ICAM-1) and vascular-cell adhesion molecule 1 (VCAM-1) (Barnes and Karin, 1997; Winyard and Blake, 1997). In addition, cellular redox status plays a role in modulating matrix metalloproteinase (MMP) activity, thereby facilitating the trafficking of T cells across the BBB and into the CNS (Leppert et al., 1995; Merrill and Murphy, 1997; Romanic and Madri, 1994).

## 3 Current therapies from an OS perspective

Although multiple disease-modifying therapies (DMTs) are currently approved for MS, most primarily target peripheral immune activation and inflammatory cell trafficking rather than directly interrupting OS-driven injury within the CNS. Among approved agents, DMF is the most closely linked to the OS context because, in addition to its immunomodulatory effects, it can enhance cellular antioxidant responses and suppress inflammatory signaling (Blair, 2019; Peng et al., 2012). In contrast, most other approved therapies may reduce oxidative damage only indirectly by attenuating inflammation, but they are not specifically designed to target persistent reactive oxygen and nitrogen species production, mitochondrial dysfunction, iron-associated oxidative injury, or chronic microglial activation. Consequently, although current DMTs effectively reduce relapse rates and inflammatory activity, ongoing neuroaxonal injury and disability progression may still occur in a subset of patients despite treatment (Dendrou et al., 2015; Jayaraman and Jayaraman, 2022; Gharibani et al., 2025). These limitations underscore the need for therapeutic strategies that more directly restore redox homeostasis and interrupt the self-perpetuating cycle between OS and inflammation in MS.

## 4 Therapeutic strategies: from traditional to novel antioxidants

Although current DMTs effectively reduce inflammatory activity in RRMS, their efficacy in progressive forms of the disease remains limited, particularly in halting the accumulation of irreversible disability driven by OS-mediated neurodegeneration. Consequently, the identification of alternative or complementary therapeutic strategies represents an important scientific objective with substantial translational potential. OS has emerged as a critical pathogenic component of MS, contributing to both inflammatory and autoimmune-mediated mechanisms of tissue injury. Within the CNS, ROS are primarily generated by activated macrophages and microglia, leading to lipid peroxidation, mitochondrial dysfunction, and axonal damage. These observations provide a strong biological rationale for antioxidant-based interventions as a therapeutic strategy aimed at slowing disease progression and limiting irreversible neurodegeneration in MS.

### 4.1 Therapeutic potential of classical antioxidants

Antioxidants play a central role in cellular defense against OS by scavenging ROS and their precursors, suppressing ROS generation, and chelating metal ions that catalyze ROS-forming reactions (Gilgun-Sherki et al., 2001). Among these defense systems, enzymatic antioxidants constitute the most efficient endogenous mechanism against ROS-mediated cellular injury (Jomova et al., 2024). SOD is a critical regulator of intracellular redox homeostasis and contributes to maintaining the balance between pro-oxidant and antioxidant processes (Li et al., 2018), it also exhibits notable anti-inflammatory properties, attenuating the production of pro-inflammatory cytokines and modulating signaling pathways such as PI3K (Phosphatidylinositol 3-kinase)/Akt (Protein kinase B)/NRF2 (nuclear factor erythroid 2-related factor 2), which coordinate antioxidant defense and inflammatory resolution (Zhang et al., 2022). CAT complements SOD activity by rapidly degrading high concentrations of H_2_O_2_ derived from both exogenous and endogenous sources (Abdalbagemohammedabdalsadeg et al., 2024), whereas GPx primarily scavenges low levels of endogenous H_2_O_2_ and effectively inhibits lipid peroxidation (Liu et al., 2023).

Although individual antioxidant enzymes are often associated with distinct catalytic functions, they typically operate in a coordinated manner within biological systems to form functional redox cascades (Ma et al., 2025). A well-characterized example is the SOD-CAT axis, in which SOD catalyzes the conversion of superoxide radicals to hydrogen peroxide, followed by CAT-mediated detoxification of hydrogen peroxide into water and oxygen. This sequential mechanism is essential for limiting oxidative damage and preserving redox balance (Yang et al., 2019).Importantly, effective intervention in complex pathological conditions such as MS is unlikely to be achieved by targeting a single antioxidant enzyme alone. Indeed, accumulating evidence indicates that combined antioxidant systems such as optimized ratios of SOD/CAT or SOD/CAT + GPx-exert superior protective effects compared with individual enzymes (Yu et al., 2007; de Haan et al., 1995).

Despite decades of intensive research aimed at the pharmaceutical development of antioxidant enzymes, particularly SOD-based therapeutics, clinical translation has remained largely unsuccessful. The development of protein- and enzyme-based antioxidant drugs faces substantial challenges that are particularly problematic for chronic diseases like MS, including immunogenicity, high manufacturing and purification costs, limited stability, poor oral bioavailability, and unfavorable pharmacokinetic profiles that preclude long-term systemic administration (Regnault et al., 1996). These formidable limitations underscore the urgent need for alternative strategies to effectively mitigate OS in MS.

### 4.2 Pharmacodynamic and pharmacokinetic challenges of antioxidant drug development for MS

Despite strong evidence implicating OS in the pathogenesis of MS, the development of antioxidant therapies has produced only limited and inconsistent clinical benefit. One major obstacle lies in the pharmacodynamic complexity of oxidative injury in MS. Reactive oxygen and nitrogen species are generated by activated microglia, infiltrating macrophages, and dysfunctional mitochondria within inflammatory lesions, where they contribute to demyelination, axonal injury, and neurodegeneration (Ohl et al., 2016). Oxidative damage in MS has been documented in both white matter and gray matter lesions and is associated with lipid peroxidation, mitochondrial abnormalities, and enhanced expression of endogenous antioxidant enzymes, underscoring that redox imbalance is a central but highly dynamic component of lesion biology (van Horssen et al., 2008). At the same time, reactive species are not solely deleterious molecules, since they also participate in physiological redox signaling and adaptive cellular responses (Tonelli et al., 2018). Accordingly, antioxidant treatment in MS cannot be reduced to simple radical scavenging; it must attenuate pathological oxidative injury without excessively interfering with redox-dependent signaling pathways that remain necessary for immune regulation, stress adaptation, and cell survival (van Horssen et al., 2010). This may partly explain why conventional antioxidants or single-mechanism agents often show encouraging preclinical effects yet fail to achieve robust therapeutic efficacy in patients.

Pharmacokinetic constraints remain a major barrier to the clinical translation of antioxidant therapies in MS. For these agents to be effective, they must retain adequate stability and bioavailability, reach CNS, and sustain sufficient exposure at sites of active neuroinflammation. In reality, however, many antioxidant compounds suffer from poor absorption, rapid clearance, limited tissue penetration, and inadequate retention, all of which compromise target engagement (Yang et al., 2019). These limitations are particularly evident in protein- and enzyme-based antioxidants. Although SOD-based approaches are attractive because of their direct ROS-scavenging activity, their development has been restricted by poor oral bioavailability, unfavorable pharmacokinetics, and limited stability (Jomova et al., 2024). Comparable formulation and delivery challenges also affect CAT- and GPx-related strategies (Abdalbagemohammedabdalsadeg et al., 2024; Liu et al., 2023). As a result, increasing attention has been directed toward enzyme engineering, immobilization, and nanozyme-based platforms to enhance stability, delivery efficiency, and *in vivo* persistence (Ma et al., 2025).

These limitations have shifted attention toward strategies that do not rely solely on exogenous radical scavengers, but instead enhance endogenous cytoprotective programs or improve delivery to diseased tissue (Lee, 2023). In this context, NRF2-centered approaches are of particular interest, because they regulate a broader antioxidant and detoxification network and may provide more sustained control of oxidative injury than conventional antioxidants with narrow mechanisms of action (Cuadrado et al., 2019).

### 4.3 Novel therapeutic strategies targeting the NRF2 pathway

#### 4.3.1 The NRF2 pathway

**FIGURE 3:**
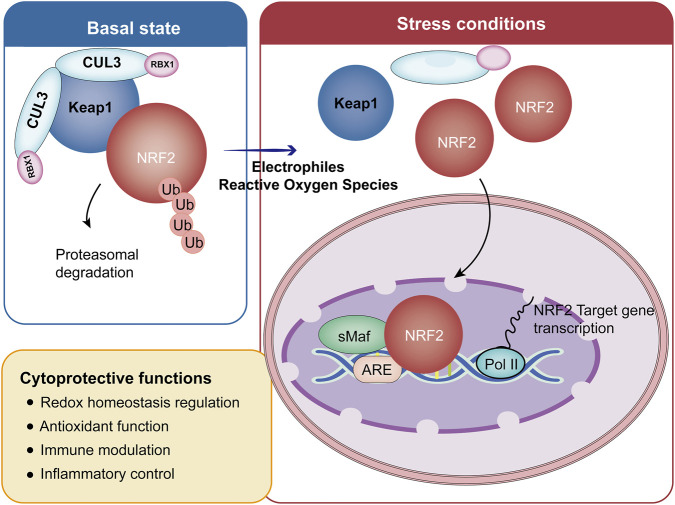
Keap1/NRF2/ARE Signaling Pathway: The Core Regulatory Mechanism of the NRF2-Mediated Cytoprotective Defense System. In the basal state, Keap1 promotes NRF2 ubiquitination and degradation. Under stress, NRF2 dissociates, translocates to the nucleus, and binds the ARE with sMaf proteins to drive transcription of cytoprotective genes. These genes regulate redox balance, antioxidant function, immune modulation, and inflammation, protecting cells from damage. CUL3, cullin-3-based ubiquitin ligase; RBX1, ring box-1 protein.

Several studies have reported dysregulation of the transcription factor NRF2, a master regulator of cellular redox homeostasis, in patients with MS and in EAE mice model (van Horssen et al., 2010; Mohajeri et al., 2015). NRF2 orchestrates the cellular response to OS by governing the expression of a broad array of cytoprotective genes under both Physiological and pathological conditions (Figure 3). The human NRF2 protein consists of 605 amino acids and contains seven highly conserved NRF2-ECH homology (Neh1-7) domains (Sahu and Jain, 2025). Among these, the NEH1 domain is responsible for binding small MAF (sMAF) proteins and interacting with antioxidant response element (ARE) binding proteins, which are conserved cis-regulatory sequence located in the promoters of various genes encoding antioxidant, detoxifying, and cytoprotective enzymes. In the nucleus, NRF2 forms a heterodimer with sMAF proteins, enabling the complex to recognize and bind to AREs and initiate the transcription of downstream target genes (Itoh et al., 1997). In contrast, the NEH2 domain mediates interaction with Keap1, a cytoplastic repressor that facilitates NRF2 ubiquitination and proteasomal degradation under basal conditions (Tong et al., 2006). Physiologically, NRF2 is predominantly sequestered in the cytoplasm through its association with Keap1, which acts as an adaptor to oxidative or electrophilic stressors, such as elevated ROS, conformational changes in Keap1 disrupt its interaction with NRF2, leading to NRF2 stabilization and release (Yamamoto et al., 2018). Stabilized NRF2 subsequently translocates into the nucleus, heterodimerizes with sMAF proteins, and binds to AREs to activate the transcription of a diverse array of target genes involved in cellular defense mechanisms (Lu et al., 2016).

The antioxidant function of NRF2 is primarily mediated through its regulation of genes controlling ROS detoxification and redox balance. These genes span several functional categories, including antioxidant defense, NADPH regeneration, detoxification and metal sequestratrion, protein turnover, and cellular maintenance processes such as DNA repair and apoptosis prevention (Ma, 2013; Hayes and Dinkova-Kostova, 2014; Kensler et al., 2007). NRF2 promotes ROS neutralization by coordinately inducing both enzymatic antioxidants-such as SOD, Prx, and GPx and non-enzymatic components, notably (GSH) (Dodson et al., 2019; Xiang et al., 2022; Done and Traustadottir, 2016). In addition, NRF2 activation upregulates key redox-regulating enzymes, including NAD(P)H: quinone oxidoreductase 1 (NQO1), heme oxygenase-1 (HO-1), sulfiredoxin-1 (SRXN1), and UDP-glucuronosyltransferase (UGT), all of which contribute to limiting OS and preserving cellular hommeostatis (Tonelli et al., 2018).

Beyond its canonical antioxidant role, the NRF2-regulated transcriptional network extends to numerous genes involved in immune modulation and inflammatory control. Following nuclear translocation, NRF2 binding to AREs initiates a protective gene program whose anti-inflammatory efficacy correlates closely with the magnitude of NRF2 activation induced by ARE-responsive compounds (Dinkova-Kostova et al., 2005; Talalay et al., 2007; Ma and Kinneer, 2002). Genetic ablation of NRF2 in mice results in heightened susceptibility to progressive multisystem pathology characterized by age-dependent autoimmune and inflammatory lesions (Yoh et al., 2001; Ma et al., 2006). Mechanistic studies have further elucidated the role of NRF-2 as a negative regulator of inflammation. Kobayashi et al. revealed that NRF2 suppresses lipopolysaccharide-induced expression of key proinflammatory cytokines, such as IL-6 and IL-1β (Kobayashi et al., 2016). In addition, NRF2 confers direct tissue protection by alleviating inflammation-driven pathological damage, at leaset in part through restraining the production of pro-inflammatory mediators (Keleku-Lukwete et al., 2015). Consistent with these findings, NRF2-deficient mice exhibit exaggerated inflammatory responses in multiple inflammation models (Itoh et al., 2004; Ishii et al., 2005). Collectively, these studies position NRF2 as a critical upstream regulator of cytokine production and inflammatory signaling, thus establishing a molecular framework for developing interventions that mediate anti-inflammatory effects through NRF2 activation. At the molecular level, NRF2-mediated anti-inflammatory effects are partly achieved through functional antagonism of the NF-κB pathway (Li et al., 2008). NRF2 activation has been shown to inhibit the phosphorylation of IKK/IκB and the nuclear translocation of p65, thus weakening the NF-κB-driven inflammatory signaling (Xu et al., 2005). Together, these findings highlight the NRF2 pathway as a compelling therapeutic target for diseases characterized by intertwined OS and inflammation, including MS.

#### 4.3.2 NRF2-targeting drugs and the therapeutic landscape

Given the expanding recognition of NRF2 as a central regulator of OS and inflammation, particularly in the context of aging and age-associated neurodegenerative disorders, positive findings from EAE models have prompted increasing interest in NRF2 modulation as a strategy to delay MS progression. A growing body of evidence suggests that antioxidant-based interventions may exert disease-modifying effects in MS, at least in part, through the activation of the NRF2 signaling pathway.

Among natural compounds, curcumin is one of the most extensively studied NRF2-activating agents in MS-related research. Curcumin possesses well-documented antioxidant and anti-inflammatory properties and has shown therapeutic potential across multiple neurodegenerative disease models (Esatbeyoglu et al., 2012). In EAE, curcumin treatment significantly ameliorates clinical severity, an effect associated with increased NRF2/HO-1 expression (Mohajeri et al., 2015), suppression of pro-inflammatory mediators, and promotion of remyelination and myelin repair (Mohajeri et al., 2015; Xie et al., 2009). However, the translational potential of curcumin is limited not only by poor aqueous solubility and low oral bioavailability, but also by rapid intestinal and hepatic metabolism, formulation-dependent exposure, and uncertain CNS delivery (Anand et al., 2007; Patel et al., 2020; Yan et al., 2025). Moreover, curcumin is pharmacologically pleiotropic rather than highly target-selective, and dose-dependent pro-oxidant effects have also been reported, underscoring the need to better define its therapeutic window in MS (Patel et al., 2020).

Resveratrol, another well-characterized natural antioxidant, exerts pleiotropic anti-inflammatory and cytoprotective effects, partly via the activation of the NRF2 signaling pathway (Shahcheraghi et al., 2023). In EAE models, resveratrol improves neurological outcomes and attenuates inflammatory responses by inducing T-cell apoptosis and reducing the production of inflammatory mediators (Shindler et al., 2010; Singh et al., 2007). However, like curcumin, challenges related to its pharmacokinetics, target specificity, and the feasibility of achieving therapeutic doses have limited its advancement into clinical application for MS. Preclinical studies often employ concentrations that would require impractically large quantities in a clinical setting, further hindering translational progress (Walle, 2011).

An independent clinically relevant association has been established between vitamin D deficiency and increased disease activity, disability progression, and functional decline in patients with MS. Administration of exogenous 1,25-(OH)_2_D_3_ suppresses EAE progression through Rag-1-dependent lymphocyte-mediated mechanisms that restrict CNS infiltration of auto-reactive T cells and macrophages, promote apoptosis of inflammatory cells, and enhance survival of resident CNS cells, an effect associated with decreased macrophage accumulation within the CNS (Nashold et al., 2001; Nashold et al., 2000). Although vitamin D has been reported to exert antioxidant effects via upregulation of NRF2 signaling pathway or antioxidant enzymes, definitive evidence demonstrating its direct efficacy in improving clinical outcomes in MS remains inconclusive (Sosa-Diaz et al., 2022). Similarly, vitamin A has been shown to alleviate EAE symptoms by suppressing key inflammatory mediators (including IL-1β, IL-12, TNF-α, and ATP) and limiting the expansion of myelin basic protein (MBP)-reactive lymphocyte populations (Sirakawin et al., 2024; Navidhamidi et al., 2022; Reza Dorosty-Motlagh et al., 2016).

Among NRF2-targeting therapies, DMF represents the most clinically advanced example in the treatment of MS. DMF is an FDA-approved first-line oral disease-modifying therapy for the treatment of relapsing forms of MS. DMF and its primary metabolite, monomethyl fumarate (MMF), are well-established activators of the NRF2 pathway, as demonstrated by consistent findings from *in vitro* systems, animal models, and clinical observations in humans. DMF stabilizes NRF2, enhances NRF2-dependent transcriptional activity, and upregulates classical target genes such as NQO1 (Linker et al., 2011). However, describing DMF solely as an NRF2 activator oversimplifies its pharmacology. DMF is increasingly recognized as a pleiotropic electrophilic molecule that can modify reactive cysteine residues in multiple proteins, thereby influencing signaling pathways beyond the canonical Keap1-NRF2 axis (Blewett et al., 2016; Schulze-Topphoff et al., 2016). In addition to enhancing antioxidant defense, DMF has been reported to modulate inflammatory signaling, immune-cell activation, and cellular metabolism through mechanisms that are at least partially independent of NRF2 (Schulze-Topphoff et al., 2016; Peng et al., 2012). Notably, accumulating evidence indicates that DMF also exerts immunomodulatory effects through NRF2-independent mechanisms. DMF has been shown to inhibit activated human Jurkat T cells through mechanisms distinct from NRF2 activation and to protect both wild-type and NRF2-deficient mice from developing acute inflammatory EAE (Kihara et al., 2015; Schulze-Topphoff et al., 2016). These findings suggest that the therapeutic efficacy of DMF in MS likely reflects a combination of NRF2-dependent antioxidant effects and parallel immunosuppressive or metabolic mechanisms. This multi-target profile has important implications for both efficacy and safety. On the one hand, pleiotropic activity may be advantageous in MS, where OS, immune dysregulation, mitochondrial dysfunction, and neuroinflammation are tightly interconnected. On the other hand, limited pathway specificity complicates mechanistic interpretation and may also contribute to adverse effects, thereby narrowing the therapeutic window in some patients (Gold et al., 2012; Naismith et al., 2020).

Results from pivotal phase III trials demonstrated that DMF significantly reduced relapse rates, delayed disability progression, and lowered MRI lesion activity in patients with RRMS (Gold et al., 2012; Fox et al., 2012). Following the clinical success of DMF, the next-generation MMF prodrug tegomil fumarate received approval from the European Medicines Agency (EMA) in July 2025 for the treatment of MS. Despite its clinical utility, DMF is associated with several limitations. After 15 months of treatment, 79.9% of patients achieved no evidence of disease activity (NEDA), defined as the absence of relapses, new MRI lesions, and disability progression, indicating that approximately 20% of patients continued to exhibit disease activity during this period (Sattarnezhad et al., 2022). In addition, DMF shows only modest efficacy in limiting long-term disability accumulation, has limited pathway specificity, and is associated with a substantial adverse-effect profile. Common and clinically significant side effects include gastrointestinal discomfort, nausea, flushing, persistent lymphopenia, and, in rare cases, treatment-associated progressive multifocal leukoencephalopathy (PML) (Naismith et al., 2020; Ermis et al., 2013; Rosenkranz et al., 2015). Collectively, these limitations support the continued evaluation of next-generation NRF2-targeting therapies designed to improve selectivity, CNS bioavailability, and safety, with the goal of determining whether such refinements can translate into incremental clinical benefit.

## 5 Discussion

Although currently approved DMTs have substantially improved the management of MS, important unmet needs remain, particularly with respect to progressive disease, neuroprotection, and long-term disability prevention. In this context, the recognized contribution of OS to MS pathophysiology supports continued investigation of redox-modulating and antioxidant-based strategies as potential future therapeutic avenues, particularly as adjunctive approaches, although their clinical value remains to be fully established. In this context, a better understanding of the interplay between inflammation and OS is of particular importance, as the mechanistic links between these processes remain incompletely defined. Current evidence indicates that ROS are major mediators of tissue injury in MS, promoting OS that disrupts the structural and functional integrity of lipids, proteins, and nucleic acids (de Vries et al., 2008). These observations support continued investigation into redox-related mechanisms as contributors to MS progression and as potential targets for adjunctive therapeutic intervention.

Under pathological conditions, OS-induced disruption of the BBB facilitates the infiltration of inflammatory cells into the CNS, where they release cytokines and other mediators that amplify neuroinflammation, creating a self-perpetuating pathological cycle. The intricate interplay between OS and neuroinflammation constitutes a fundamental driver of disease progression in MS, as well as in other autoimmune and neurodegenerative disorders of the nervous system (Absinta et al., 2021). Effective antioxidant interventions must therefore be tailored to the specific characteristics of OS-such as ROS species, cellular sources, and injury severity-and possess sufficient BBB permeability to achieve therapeutic concentrations within the CNS. Moreover, therapeutic efficacy may be further limited by the challenge of compartmentalized CNS inflammation that systemic antioxidants may not reach, particularly in progressive MS, where inflammatory processes can become spatially restricted behind an intact or only partially disrupted BBB. Consequently, the development of safe and effective pharmacological agents that integrate antioxidant capacity with immunomodulatory activity represents a promising and strategically important direction for future MS therapies.

As a master transcriptional regulator of cytoprotective responses, NRF2 plays a critical role in mediating neuroprotection through its coordination of antioxidant defenses, regulation of inflammatory signaling, and maintenance of cellular redox homeostasis (Tonelli et al., 2018). Substantial evidence from both *in vivo* and *in vitro* experimental models indicates that NRF2 activation protects neural cells against oxidative damage induced by diverse stressors (Dinkova-Kostova and Copple, 2023; Cuadrado et al., 2020). In the CNS of individuals with MS, NRF2 and its canonical downstream targets, including HO-1 and NQO-1, are markedly upregulated within active demyelinating lesions and their surrounding areas. This localized induction appears to represent a compensatory cellular response aimed at counteracting excessive OS (Licht-Mayer et al., 2015; van Horssen et al., 2010). Activation of NRF2 has been shown to exert protective effects across a broad spectrum of diseases, including autoimmune conditions such as MS (Cuadrado et al., 2019). A persistent limitation in combating oxidative damage in MS, however, lies in the insufficient endogenous activation of the NRF2 signaling cascade under pathological conditions (Tonev and Momchilova, 2023). Genetic studies have demonstrated that enhanced NRF2 expression significantly attenuates disease progression in EAE models (Kobayashi et al., 2016). Consequently, numerous small-molecule compounds capable of activating NRF2 have been developed. Notably, so-called “NRF2 activators” primarily function as inhibitors of Keap1, the cytoplasmic repressor that targets NRF2 for proteasomal degradation (Magesh et al., 2012). Clinically relevant NRF2-modulating agents currently include MMF (approved for relapsing forms of MS), diroximel fumarate (approved for relapsing forms of MS), tegomil fumarate (approved for RRMS by the EMA in 2025), and omaveloxolone (approved for Friedreich’s ataxia; under investigation for MS) (Lee, 2023). In addition, widely studied experimental NRF2 inducers encompass natural and synthetic compounds such as sulforaphane (SFN) (Ma et al., 2023), bardoxolone methyl (CDDO-Me) (Abed et al., 2015), and curcumin (Patel et al., 2020).

Importantly, NRF2 activity is tightly regulated in a spatially and temporally dependent manner to ensure appropriate cytoprotective responses (Xue et al., 2015). Nevertheless, NRF2 activation may also have a narrow therapeutic window. While NRF2 activation is generally considered protective in the context of OS and neuroinflammation, its therapeutic modulation should not be viewed as uniformly beneficial. Increasing evidence indicates that chronic or excessive NRF2 activation may, in certain biological contexts, promote tumor cell survival, metabolic reprogramming, and resistance to stress, thereby narrowing the therapeutic window of NRF2-directed interventions (Cuadrado et al., 2019; Tonelli et al., 2018; Sahu and Jain, 2025). This consideration further underscores the importance of defining the therapeutic window of NRF2 modulators in MS (Dinkova-Kostova and Copple, 2023). Therefore, in MS, the efficacy of NRF2-targeting strategies is likely to depend not only on pathway activation *per se*, but also on the magnitude, duration, and cellular context of activation. A more precise definition of target engagement and dose-response relationships will be important to maximize benefit while minimizing potential risks associated with NRF2 overactivation (Jin et al., 2025; Mathis et al., 2022).

The limited and inconsistent clinical efficacy of antioxidant strategies in MS likely reflects, at least in part, pharmacological constraints that are not captured by preclinical efficacy alone (Reich et al., 2018). Many candidate compounds (such as curcumin) have poor oral bioavailability, rapid metabolism, formulation-dependent exposure, and uncertain CNS penetration, while plasma pharmacokinetics may not accurately represent drug levels within compartmentalized CNS lesions, especially in progressive disease (Liu et al., 2016; Rankovic, 2015; Sweeney et al., 2019; Lassmann, 2018). In addition, the marked pleiotropy of many redox-active agents complicates mechanistic attribution and makes it difficult to separate target-dependent effects from broader off-target actions (Robledinos-Anton et al., 2019; Cuadrado et al., 2018). These issues may collectively explain why promising experimental findings have translated only weakly or inconsistently into clinical benefit, and they underscore the importance of improved brain delivery, pharmacokinetic-pharmacodynamic integration, and target-engagement biomarkers in future MS drug development (Sweeney et al., 2019; Morgan et al., 2012).

Nevertheless, the therapeutic implications of antioxidant strategies in MS should be interpreted cautiously. OS is not a uniform pathological process, but a context-dependent event influenced by reactive species type, cellular source, lesion stage, and the surrounding inflammatory milieu. Because reactive oxygen and nitrogen species also participate in physiological signaling and cellular adaptation, broad suppression of oxidative pathways may not always be beneficial. Accordingly, antioxidant interventions are unlikely to be universally effective, and their benefit may depend on selective modulation of pathological redox imbalance. In addition, despite the mechanistic appeal of antioxidant therapy, its translation remains constrained by major pharmacological barriers, including poor stability, limited bioavailability, suboptimal pharmacokinetics, and inadequate CNS delivery, especially for protein- and enzyme-based antioxidants. Although endogenous pathway–oriented approaches such as NRF2 activation may offer broader cytoprotective effects, issues related to selectivity, treatment timing, safety, and CNS exposure remain unresolved. Therefore, future therapeutic development should prioritize more targeted and pharmacologically optimized strategies capable of interrupting the OS-inflammation vicious cycle without compromising physiological redox homeostasis. These limitations do not diminish the importance of OS in MS, but rather emphasize the need for more selective and pharmacologically optimized therapeutic approaches.

## 6 Future directions

Despite growing evidence supporting the role of OS in MS, several important questions remain unresolved. First, the mechanistic links between neuroinflammation and oxidative damage require further clarification, particularly across different lesion stages and disease phenotypes. Second, clinically useful biomarkers of redox imbalance are still lacking, limiting patient stratification and treatment monitoring. Third, future therapeutic development should focus on restoring redox homeostasis without disrupting physiological ROS-dependent signaling, rather than relying on indiscriminate antioxidant approaches. Finally, given the strong interaction between immune dysregulation and oxidative injury in MS, combination strategies integrating antioxidant and immunomodulatory effects may represent a more effective direction for future translational and clinical research.
